# Altered Purinergic Signaling in Neurodevelopmental Disorders: Focus on P2 Receptors

**DOI:** 10.3390/biom13050856

**Published:** 2023-05-18

**Authors:** Marta Boccazzi, Stefano Raffaele, Thomas Zanettin, Maria P. Abbracchio, Marta Fumagalli

**Affiliations:** 1 Laboratory of Molecular and Cellular Pharmacology of Purinergic Transmission, Department of Pharmaceutical Sciences, Università Degli Studi di Milano, 20133 Milan, Italy; marta.boccazzi@unimi.it; 2 Laboratory of Molecular and Cellular Pharmacology of Purinergic Transmission, Department of Pharmacological and Biomolecular Sciences, Università Degli Studi di Milano, 20133 Milan, Italy; stefano.raffaele@unimi.it (S.R.); marta.fumagalli@unimi.it (M.F.)

**Keywords:** purinergic signaling, P2 receptors, neurodevelopmental disorders, maternal immune activation

## Abstract

With the umbrella term ‘neurodevelopmental disorders’ (NDDs) we refer to a plethora of congenital pathological conditions generally connected with cognitive, social behavior, and sensory/motor alterations. Among the possible causes, gestational and perinatal insults have been demonstrated to interfere with the physiological processes necessary for the proper development of fetal brain cytoarchitecture and functionality. In recent years, several genetic disorders caused by mutations in key enzymes involved in purine metabolism have been associated with autism-like behavioral outcomes. Further analysis revealed dysregulated purine and pyrimidine levels in the biofluids of subjects with other NDDs. Moreover, the pharmacological blockade of specific purinergic pathways reversed the cognitive and behavioral defects caused by maternal immune activation, a validated and now extensively used rodent model for NDDs. Furthermore, Fragile X and Rett syndrome transgenic animal models as well as models of premature birth, have been successfully utilized to investigate purinergic signaling as a potential pharmacological target for these diseases. In this review, we examine results on the role of the P2 receptor signaling in the etiopathogenesis of NDDs. On this basis, we discuss how this evidence could be exploited to develop more receptor-specific ligands for future therapeutic interventions and novel prognostic markers for the early detection of these conditions.

## 1. Introduction

Neurodevelopmental disorders (NDDs) comprise a wide variety of neuropathological conditions related to alterations of brain development. These broadly include autism spectrum disorders (ASDs), schizophrenia (SCZ), attention deficit hyperactivity disorder (ADHD), intellectual disability disorder, Rett syndrome, Down syndrome, cerebral palsy and Williams Syndrome, as well as many other rare genetic disorders such as Lesch–Nyhan disease (LND) [1]. Despite very heterogeneous clinical causes, which involve either genetic or gestational/perinatal insults, all NDDs are characterized by a combination of cognitive, social, and motor deficits which manifest early in childhood and require life-long healthcare assistance, implicating a severe socio-economic burden [2]. NDDs affect >3% of children worldwide, and their incidence is expected to dramatically increase in the next decade due to the consequences of gestational infections during the COVID-19 pandemic [3]. In this scenario, identifying novel therapeutic targets to tackle the disability associated with NDDs represents an urgent medical need.

Proper neural development involves a time-dependent orchestrated sequence of genetic, epigenetic, and environmental events that are crucial for shaping the architecture of the growing brain. Synapse formation, the functionality of neural networks and the myelination of neuronal axons require a precise fulfillment of multiple developmental processes including cell fate specification, cell migration, axon guidance and specific cell death programs in damaged or unnecessary cells [4,5].

In recent years, solid evidence acknowledged a pivotal role of purinergic signaling, constituted by adenosine which activates the G protein-coupled P1 receptors (A_1_, A_2A_, A_2B_ and A_3_) [6] and ATP and other extracellular nucleotides interacting with the ligand-gated P2X channels (the P2X1-7 subtypes) and G protein-coupled P2Y receptors (the P2Y_1,2,4,6,11,12,13,14_ subtypes) [7] in orchestrating fetal and postnatal brain development [8]. For instance, adenosine signaling has been shown to dramatically affect neural plasticity [9] and, through the A_2A_ receptor subtype, to promote neurite outgrowth [10] and to control neuronal migration and wiring during development [11,12,13,14]. Moreover, adenosine is known to act as a key regulator of oligodendrocyte precursor cell (OPC) maturation [15]. On the other hand, the expression of specific P2 receptor subtypes has been shown to be highly dynamic and tightly controlled at different developmental stages, correlating with a specific temporal pattern required to control brain maturation [16]. For instance, P2Y_1_ receptor-mediated calcium signaling is fundamental to guiding the migration of neural precursors to developing cortical layers and their differentiation in neurons or astrocytes [17]. In addition, microglia, which are known to colonize the brain very early during fetal development, were recently shown to interact with the soma of immature neuronal precursors by forming specialized interaction sites enriched in P2Y_12_ receptors, defined as somatic purinergic junctions, which are essential to driving neuronal proliferation during cortical development [18]. The purinergic system is crucially involved also in the later stages of neuronal development, with purine P2Y_1_,_13_ and pyrimidine P2Y_2_ receptor subtypes regulating neurite elongation and astrocyte terminal maturation [10,19]. Furthermore, the activity-dependent extracellular release of ATP is one of the main signaling mechanisms shaping axonal myelination by activating several P2 receptors expressed on OPCs, which regulate their proliferation, migration and differentiation into mature oligodendrocytes during early postnatal development [20,21]. Finally, P2Y and P2X receptor subtypes have been detected in adult neurogenic niches, the ventricular/subventricular zone and the subgranular layer of the hippocampus, where they modulate neural precursor cell functions during postnatal development and injury-induced brain remodeling [22,23,24]. In particular, P2X7 receptor expression is necessary to maintain neural stem cells in a mitotic state required for self-renewal and injury response, while it was found to decrease throughout neurogenesis to allow axonal elongation and branching [25]. Accordingly, *P2X7* knockout mice displayed defects in neuronal differentiation and dendritic spines [26].

Given the importance of the purinergic system for proper brain development, it is not surprising that alterations of purine metabolism and signaling cascades have been involved in the pathogenesis of many NDDs. As the contribution of adenosine signaling to neurodevelopmental disorders has been exhaustively and elegantly dissected elsewhere [8,27,28,29,30], here, we focus on the role of P2 signaling during early pathological events representing possible triggers of NDDs, such as maternal immune challenge, perinatal injury and genetic alterations, with the final aim of inspiring purinergic therapeutic interventions able to halt the progression of these devastating conditions.

## 2. Clinical Evidence Linking Neurodevelopmental Disorders with Alterations of Purinergic Signaling and Purine Metabolism

Numerous clinical studies have associated NDDs with errors in purine and pyrimidine metabolism. Purine metabolism is divided into three pathways: the de novo biosynthetic pathway, which generates inosine monophosphate (IMP); the catabolic pathway, which generates uric acid; and the salvage pathway, which reconverts guanine, hypoxanthine and adenine into guanosine monophosphate (GMP), IMP and adenosine monophosphate (AMP), respectively [31]. 

The first described syndrome linked to abnormal purine biosynthesis was the Lesch–Nyhan disease (LND) [32]. LND is an X-linked genetic disorder caused by mutations of the *HPRT1* gene encoding for the hypoxanthineguanine phosphoribosyltransferase (HPRT) enzyme, which catalyzes the conversion of hypoxanthine and guanine to IMP and guanosine monophosphate [31]. *HPRT* deficiency leads to increased de novo purine synthesis, resulting in elevated levels of uric acid that can precipitate in body fluids with subsequent hyperuricemia, nephrolithiasis and gout [33]. Besides an aberrant purine metabolism, the syndrome is characterized by deficient basal ganglia dopamine levels, dystonia and severe neurobehavioral manifestations, including compulsive self-injurious behavior. Furthermore, a 3-year-old boy displaying autistic behaviors and impaired hearing associated with a flaw in purine biosynthesis was described in 1969 by William Nyhan. In this child, a mutated form of phosphoribosyl pyrophosphate synthase, an enzyme responsible for the first committed step in purine biosynthesis, was found to be resistant to adenosine triphosphate’s (ATP) negative feedback, thus causing the enzyme to be abnormally active and inducing, in turn, an excessive biosynthesis of purines [34]. Subsequently, changes in the purine and pyrimidine metabolite profile have been extensively described in human biofluids of children with ASD [35,36,37,38,39]. A very recent study combined metabolomics and transcriptomics analysis to verify purine metabolism dysfunction in children affected by ASD and to identify potential biomarkers [40]. Through metabolomics analysis on plasma, authors confirmed that the purine metabolic pathway was significantly altered in patients with ASDs and found that uric acid was one of the most significant differential metabolites between ASDs and controls. RNA-seq analysis revealed significant differences between the two groups in the transcriptional expression of two enzymes involved in the last steps of purine de novo biosynthesis, the adenosylosuccinate lyase (ADSL) and AICAR transformylase/IMP cyclohydrolase (ATIC), and of the adenosine catabolizing enzyme adenosine deaminase (ADA) [40]. These examples suggest that alterations in purine biosynthesis and catabolism may contribute to neurological outcomes. However, it is not clear whether these changes affect the concentration of extracellular purine nucleotides in body fluids. Although most of these pathways have been shown to influence adenosine availability and, consequently, its signaling [8,41], a paper published last year found consistently elevated levels of serum ATP and ADP in acutely hospitalized schizophrenic patients compared to controls. Moreover, ATP and ADP were significantly positively correlated with the Positive and Negative Symptom Scale item “lack of judgment and insight”, thus suggesting a potential relationship between blood ATP levels and disease activity [42]. Nevertheless, it is worth clarifying that increased nucleotide levels in peripheral biofluids do not necessarily mirror purinergic alterations occurring in the CNS and may simply reflect the response of circulating immune cells. On this basis, specific studies investigating the pathophysiological correlation between purine levels in the serum and brain parenchyma and disease severity are certainly encouraged.

Furthermore, evidence of altered expression of several P2 receptors has been reported. In particular, the expression of *P2Y_2_* and *P2Y_6_* resulted in significant upregulation, while the expression of *P2X7* was significantly downregulated in the blood of ASD children compared to healthy subjects [40]. In addition, *P2X7*, *P2X4* and *P2X5* mRNA expression was significantly increased in the dorsolateral prefrontal cortex of subjects diagnosed with SCZ compared to matched, non-psychiatrically ill controls. P2X7 was also significantly increased at protein levels [43]. 

**Figure 1:**
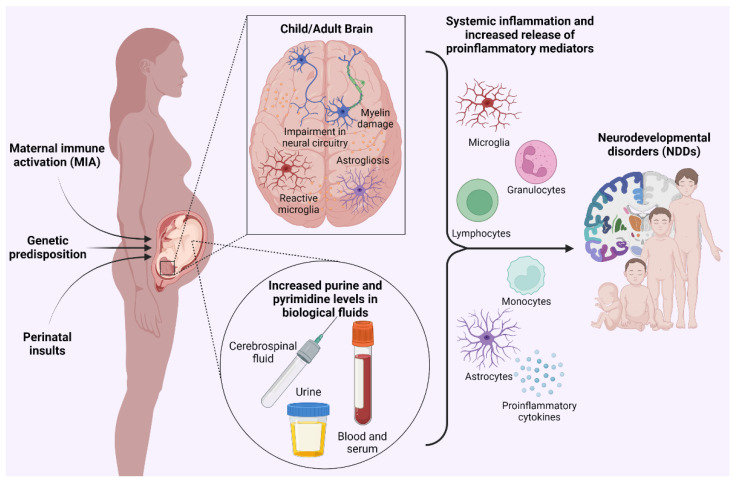
During pregnancy, perinatal insults and maternal immune activation (MIA), together with genetic predisposition, may compromise the physiological development of fetal brains’ cytoarchitecture, which is then mirrored by impairments in the formation of neural circuitries, astrogliosis, microglia shifting from a homeostatic to a reactive state and myelin damage in the brain of affected subjects during postnatal life. These pathological alterations have also been reported to be paralleled by changes in the purine and pyrimidine metabolites profile, as detected in human biofluids of children affected by neurodevelopmental disorders (NDDs). Epidemiological studies support the theory that a state of systemic inflammation and the substantial release of proinflammatory mediators may be related to the increased levels of purines detected in patients suffering from NDDs, which may act as damage-associated molecular patterns (DAMPs) and, thereby, potentially lead to defects in the physiological development of the central nervous system (CNS).

In conclusion, based on the well-documented role of ATP as a danger signal [44,45], the data presented above support the hypothesis that, in NDDs, increased extracellular nucleotide levels act as damage-associated molecular patterns (DAMPs), ultimately leading to an inflammatory response which may potentially interfere with the normal development of the central nervous system (CNS; Figure 1) [46]. 

## 3. Therapeutic Potential of P2X7 Receptor in Maternal Immune Activation (MIA)

Epidemiological studies have revealed that numerous pathogens affecting the maternal compartment are responsible for interfering with the correct development of the fetal brain, including a wide range of viral, bacterial and protozoan infections. In this respect, a more elevated incidence of schizophrenic and autistic newborns has been attributed to seasonal outbreaks and epidemics of rubella, influenza, measles, mumps and poliomyelitis [47]. Although prenatal infections do not per se necessarily induce clinical manifestations in humans [48,49], preclinical animal models have demonstrated that maternal immune activation (MIA) following infections plays a key role in neurodevelopment disorders, since it leads to a cascade of events which negatively affect the crucial processes involved into the formation of the growing fetus’ brain architecture [50]. Thus, MIA has been proposed to act as a “disease primer” [51,52], and several animal models of MIA have been established to investigate the mechanisms causally linking prenatal immune activation with neurochemical and behavioral changes in adult MIA offspring [53,54]. 

Physiologically, during embryonic development, a specific balance of cytokines constitutively expressed between maternal and fetal compartments is established [55]. In addition, being mammals, both humans and rodents possess a hemochorial placenta, which is permissive for direct contact between mother and fetus; this organ is responsible for strict control over the exchange of cytokines between these two environments [56]. When a pathogen or maternal cytokines and chemokines excessively permeate the fetal environment, as in the case of MIA, the embryo’s immune system is not developed enough to adequately respond to a higher amount of proinflammatory mediators, which then leads to impairments in the molecular, structural and functional integrity of the developing CNS and, eventually, to life-lasting neurological defects [57]. Models of MIA have been shown to display behavioral deficits and brain abnormalities typical of some major neurodevelopmental disorders and psychiatric illnesses, such as schizophrenia and ASDs [58]. 

In recent years, the use of polyinosinic–polycytidylic acid (Poly(I:C)) for the induction of MIA in animal models has been widely established in the scientific community (for an extensive review [59]). In this type of model, pregnant dams are exposed to an immunogenic stimulus by single or multiple intraperitoneal injections of Poly(I:C) at specific times of gestation. Poly(I:C) is a commercially available synthetic analog of double-stranded RNA (dsRNA) which activates Toll-like receptor 3 (TLR3), leading to the production and release of numerous proinflammatory mediators in the extracellular environment. The production of these cytokines by Poly(I:C) is fundamental to mimicking the acute phase of a viral infection and the influences that a viral pathogen may have on the development of the fetal brain [60]. 

Recent studies have investigated the possibility of exploiting purinergic signaling as a target for pharmacological therapy in an MIA model of ASDs. In the first study, Naviaux et al. administered a double intraperitoneal dose of Poly(I:C) to C57BL/6J pregnant dams, a first dose of 3.0 mg/kg at E12.5 and a second dose of 1.5 mg/kg at E17.5. Contemporarily, an injection of 5 μL/g phosphate-buffered saline (PBS) was intra-peritoneally (i.p.) administered on the same days to produce control offspring [61]. Afterwards, when male offspring reached 6 weeks of age, animals were treated with weekly injections of either PBS (5 μL/g, i.p.) or suramin (hexasodium salt, 20 mg/kg, i.p.), a broadly acting P2 receptor antagonist. Finally, at 8 weeks of age, all animals were evaluated by a series of test paradigms. 

In this protocol, defects in the social preference test and in the sensorimotor coordination were observed, which were accompanied by a reduction in the number of cerebellar Purkinje cells. Interestingly, behavioral and structural brain abnormalities were corrected by suramin. In addition, in the MIA offspring, the authors reported ultrastructural synaptic dysmorphology and a reduction in the expression of P2Y_2_ and P2X7 receptors and of their key effectors, extracellular signal-regulated kinases 1/2 (ERK1/2) and calcium/calmodulin-dependent protein kinase (CAM). Authors suggested that, at least for P2Y_2_, the downregulation of the receptor was due to chronically elevated purinergic signaling in the Poly(I:C) model, which is consistent with the hypothesis that excessive purinergic signaling is a causal factor that initiates and maintains the cellular danger response in the MIA model of ASDs. Of note, treatment with suramin was also able to revert the abovementioned molecular and neuropathological defects. Finally, suramin treatment also restored body temperature and brain mitochondrial activity to normal and increased whole-body oxygen consumption (metabolic rate, VO2) in MIA animals [61]. 

In a subsequent study of the same group [46], MIA was induced in the same way, but male offspring were treated with a single injection of either PBS or suramin when they reached 26 weeks of age (almost 6 months), equivalent to a human biological age of 30 years. The results of behavioral tests evidenced again that social and novelty preferences were deficient in MIA animals treated with PBS, whereas these deficits were completely reversed in animals following the single suramin injection. It has also been proven that, after 5 weeks of drug washout, no benefit remained concerning the novelty preference, whereas a small residual social preference benefit persisted. Different than seen before, a single dose of suramin did not restore normal motor performance. However, motor coordination is critically dependent on the integrity of Purkinje cells, which are known to be lost in MIA animals by 4 months (16 weeks) of age. Thus, it could well be that therapy with P2 receptor antagonists given later in life (at or after 6 months of age) would have no effect on Purkinje cell loss and ultimately on motor performance. According to metabolomic analyses, purine metabolism was the most affected metabolic pathway in MIA mice; 23% (11 out of 48) of the discriminant metabolites were purines, and 82% (9 out of 11) of purine metabolites were increased in untreated MIA mice, consistent with the condition of excessive purinergic signaling. This pattern of abnormalities is strikingly comparable to the metabolic abnormalities that have been found in ASD children. Suramin restored the level of the nine dysregulated purine metabolites in plasma to more normal levels [46]. Furthermore, this experiment allowed us to understand how drugs that cannot permeate the blood–brain barrier (BBB) can still have an effect in the CNS. In fact, suramin cannot pass through the BBB, but it is taken up by cells of a brain region containing a critical density of chemosensory neurons, the area postrema of the brainstem, which is not protected by the BBB. 

**Figure 2:**
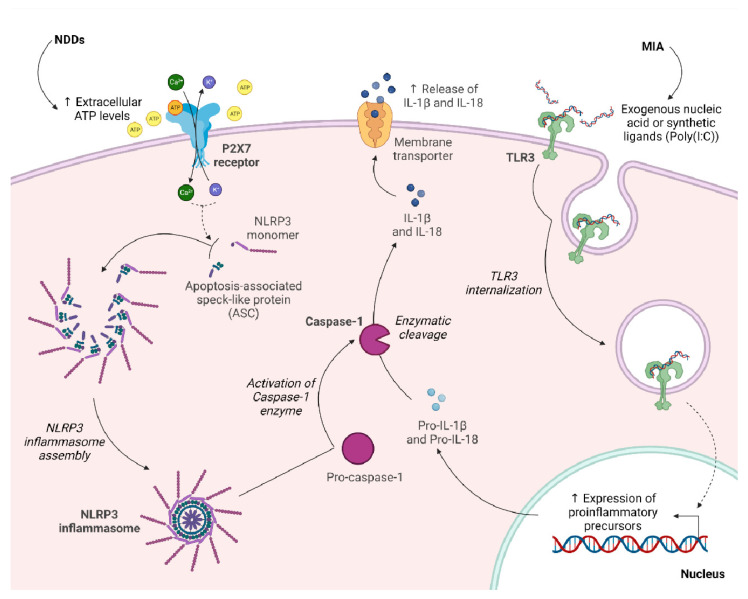
Schematic representation of the P2X7-NLRP3 and TLR3 pathways of innate immune response following an inflammatory insult. Extracellular ATP derived from damaged cells activates the P2X7 purinergic receptor, resulting in a decrease in intracellular K^+^ and increase in intracellular Ca^2+^. These molecular events lead to the oligomerization of NLRP3 subunits and to the recruitment and oligomerization of the apoptosis-associated speck-like protein (ASC), which contains the caspase recruitment and activation domain (CARD), giving rise to the mature NLRP3 inflammasome. Subsequently, ASC filaments recruit pro-caspase-1 to the NLRP3 inflammasome and promote its activation into caspase-1. Concurrently, the transmembrane Toll-like receptor 3 (TLR3) senses the presence of exogenous nucleic acids or synthetic immune stimulants, such as Poly(I:C), in the extracellular environment, and, upon activation, it is internalized. TLR3 internalization leads to increased production of proinflammatory precursors, particularly pro-IL-1β and pro-IL-18, which are then cleaved by caspase-1 into their mature forms, namely, IL-1β and IL-18. Mature proinflammatory mediators are then released in the extracellular environment. An upward pointing arrow means an increase, whereas a downward pointing arrow is indicative of a decrease.

In the wake of these encouraging results, several more studies point to maternal and offspring P2 receptors as potential therapeutic targets for the early prevention and treatment of MIA-induced mood disorders [62]. Among various P2 receptors, the P2X7 subtype received particular attention, based on its known activity in regulating neurotransmitters’ release (e.g., glutamate and GABA release [63]) in the CNS by the modulation of Ca^2+^ influx. Moreover, it has been demonstrated that P2X7 activation plays a role in the processing and release of mature proinflammatory mediators, including IL-1β, IL-18, TNF-α and ATP-mediated apoptosis. DAMPs and PAMPs (pathogen-associated molecular patterns) activate and stimulate TLRs in the production of proinflammatory cytokines precursors, such as pro-IL-1β and pro-IL-18. In parallel, after an immune challenge, cell and tissue damage leads extracellular ATP levels to increase, thus activating P2X7 receptors [64]. As a consequence, the assembly of NLRP3 inflammasome, a multimeric component of the innate immune system involved in the initiation of inflammatory cell death and proinflammatory mediators’ production, occurs, which in turn induces the activation of caspase-1. The caspase-1 enzyme is then responsible for the activation and release of mature IL-1β and IL-18. The P2X7-NLRP3 pathway is thereby crucial to the link between innate immune response and inflammation [65] (Figure 2).

In a compelling study, Horváth et al. investigated whether P2X7R activation is a necessary step in the transduction of MIA into the development of ASDs in mouse offspring. They observed that Poly(I:C) intraperitoneal injections of 3 mg/kg on E12.5 and 1.5 mg/kg on E17.5 did not induce behavioral abnormalities in the P2X7 receptors KO mouse model; furthermore, the atrophy of cerebellar Purkinje cells and malformations of synaptosomes were found to be alleviated or totally absent [66]. 

Interestingly, similar outcomes have been observed when a single injection of the potent and selective P2X7 receptor antagonist JNJ47965567 (30 mg/kg i.p.) was administered to pregnant WT dams 2 h before the Poly(I:C) administration, or postnatally to the offspring before the first behavior test. In addition, to underline the fundamental role of the P2X7 receptor in ASD development, the authors induced P2X7 receptor activation via a single ATP injection in both WT and KO mice. P2X7 receptor WT mice displayed symptoms comparable to the ones observed in Poly(I:C)-induced MIA, while P2X7 receptor KO mice did not display any deficit, thereby further supporting the hypothesis that P2X7R activation is necessary and sufficient to induce an autistic phenotype in mouse models [66]. In another publication from the same group [67], to further validate P2X7R as a potential drug target, JNJ47965567 was administered accordingly to a paradigm which was more similar to a potential human therapeutic treatment period. Indeed, MIA offspring were intraperitoneally injected for nine days starting from postnatal day (P) 65, 2 h before the respective behavior tests. Of note, JNJ47965567 was effective in the prevention of autism-like behavior in mice also using this repeated dosing protocol. In addition, given the pivotal role of NLRP3 in the innate immune response and its involvement in P2X7-mediated actions, the effect of maternal pretreatment with its selective antagonist MCC950 was investigated in the same MIA model. Autism behavior and proinflammatory mediator increases were also not observed in MIA offspring after the administration of a selective NLRP3 inhibitor, which closes the active enzyme conformation to the inactive state, confirming, once again, that the P2X7-NLRP3 pathway is necessary for the expression of the autistic phenotype [67]. 

Finally, in a very recent paper, it has been shown for the first time that MIA induced by a single dose of Poly(I:C) at E12.5 compromised dendritic outgrowth in hippocampal neurons and led to specific schizophrenia-like behaviors in a P2X7R-mediated manner. Indeed, the ablation of the receptor counteracted these structural brain abnormalities and prevented behavioral alterations. However, the effects of P2X7 receptor antagonists on these parameters have not yet been investigated [68].

### Clinical Study

Notwithstanding the fact that suramin is a rather unspecific drug and presents some substantial drawbacks regarding the extension of its use from mice to humans [62], small phase I and II clinical trials have been performed on children with ASD [69]. The Suramin Autism Treatment-1 (SAT-1) clinical trial (NCT02508259) is a double-blind, placebo-controlled, translational pilot study aimed at determining the safety, the pharmacokinetics and the pharmacodynamics of low doses of suramin in children affected by ASDs. Ten male patients, aged 5 to 14, were divided into five pairs according to their age, IQ, and degree of autism. They were then randomly assigned to receive either a single intravenous infusion of suramin (20 mg/kg) or saline solution. Purine metabolism was the pathway most altered by suramin treatment in children with ASDs, according to the pharmacometabolomic analyses and consistent with the results obtained by preclinical mouse models [46]. Following a single low dose of suramin, parents reported that the rate of new behavioral and developmental improvements continued to rise for the first three weeks after the administration, as the drug’s levels in the plasma fell from 12 to 4 μmol/L. Thereafter, these improvements gradually decreased toward baseline over the following three weeks, as plasma levels further dropped from 4 to 1.5 μmol/L. This response pattern thereby showed a threshold effect at around 4 μmol/L. In conclusion, this small trial demonstrated that low-dose suramin is a safe and promising pharmacological therapy for ASDs [69].

## 4. Involvement of Purinergic Transmission in Perinatal White Matter Injuries

A leading cause of perinatal brain injury is preterm birth, defined by the World Health Organization (WHO) as a birth before 37 completed weeks of gestation. Although the specific cause is still unknown, a multitude of overlapping factors, e.g., increases in maternal age, exposure to environmental contaminants, changes in systemic inflammatory and immune processes, social inequity and exposure to stress, have been considered risk factors for premature births [70]. Data show that 11% of live births in the world are the result of preterm delivery [71]. Outcomes of preterm birth range from subclinical psychological traits, such as mild attention deficit, to lifelong neurological disorders, such as cerebral palsy. Prematurity is also a significant risk factor for neurodevelopmental disorders such as ADHD and ASDs [72]. Diffuse perinatal white matter injuries (WMI), including periventricular leukomalacia (PVL) caused by ischemic damage, are the most common types of brain injury in preterm infants. Indeed, in humans, myelination starts only after 32 weeks of gestation and gradually increases (and is completed) within the first two decades of life. The formation of myelin is a complex process, during which OPCs become mature OLs through a highly regulated program of differentiation. The extraordinary metabolic demands and the complexity of oligodendrogenesis render this neurodevelopmental stage very vulnerable [73]. Based on the known pathophysiological role of purines in neurodevelopment [8], the presence of perinatal white matter abnormalities may also reflect alterations of specific purinergic signaling pathways. Of note, in an in vivo model of ischemic-induced PVL established by the ligation of bilateral common carotid arteries, followed by 30 min of exposure to hypoxia in P2 rats, intraperitoneal injection of uridine-5’-diphosphate (UDP)-glucose resulted in an overall amelioration of the prognosis. UDP–glucose is an endogenous agonist acting on different purinergic receptor subtypes, including the G protein-coupled P2Y-like receptor GPR17 [74]. UDP-glucose significantly improved the developmentally delayed age of first eye opening associated with hypoxic/ischemia-induced PVL. In addition, purinergic treatment reduced the white matter damage in the internal capsule, subcortical area and corpus callosum observed in the PVL animals, at 7 days and 21 days after injury. Finally, UDP–glucose improved limb motor dysfunction and incoordination, spatial identification, learning and memory functions in rats after ischemic white matter injury of PVL [75]. As a mechanism of action, it has been suggested that UDP–glucose stimulated the proliferation of glial progenitor cells derived from both the ventricular/subventricular zone and white matter, promoted their differentiation into mature myelinating oligodendrocytes and raised the survival rate of newly generated glial cells [76]. The latter results are apparently in contradiction to a recent report which used a different model of PVL established in 2-day-old neonatal rats by intracerebral injection of lipopolysaccharide (LPS) [77]. Compared to sham, GPR17 was significantly upregulated in the PVL group at 1, 3 and 7 days post-modeling. In addition, counteracting its pathological increase by in vivo silencing induced a dramatic increase in the formation of myelin sheaths as well as thicker myelin sheaths on day 7 post-modeling in the PVL + siRNA-GPR17 group with respect to PVL [77]. 

In recent years, particular importance has been gained by the interleukin-1β (IL-1β) animal model of preterm birth. Specifically, intraperitoneal injections of IL-1β (10 mg/kg) administered to mouse pups twice daily on postnatal days 1 to 4 and once on postnatal day 5 recapitulate the systemic inflammatory insult of maternal/fetal infections in a developmental period roughly corresponding to 22–32 weeks of human gestation. This, in turn, induces central inflammation, structural alterations in the brain and behavioral defects. In particular, an oligodendrocyte maturation delay can be observed, which is reflected by an increase in the numbers of NG2-positive and PDGFRα-positive OPC/pre-oligodendrocyte populations, a decreased expression of MBP, MAG and MOG and altered axonal myelination in adulthood associated with cognitive dysfunction [78]. 

Recently, in the same animal model, differences in the sensitivity to neuroinflammation between OPCs and immature OLs have also been highlighted. Purified O4-positive immature OLs showed a greater upregulation of Toll-like receptor 3 (*Tlr3*), Il-1β, interferon (*Ifn*)-β, *Ccl2* and *Cxcl10* as compared to PDGFRα-positive sorted OPCs [79]. In this model, treatment with the anti-asthmatic drug montelukast (MTK), an antagonist of both cysteinyl-leukotrienes receptor 1 (CysLTR-1) and GPR17 (see also below) has been shown to produce beneficial effects on brain abnormalities and functional deficits when concomitantly injected with IL-1β. In detail, MTK attenuated both peripheral and central inflammation, reducing the expression of pro-inflammatory molecules (IL-1β, IL-6, TNF) in the brain, restoring parvalbumin-positive interneuron density in the cortex and improving anxiety and spatial learning deficits in this model of perinatal inflammatory injury. Of note, montelukast has been also demonstrated to antagonize GPR17 [80,81,82] (see also below) to promote white matter repair in a CysLTR-1 independent way by inhibiting the receptor [83,84] and to restore cognitive function in old mice via a GPR17-dependent mechanism [85]. Moreover, functional genomic analyses highlighted a shift in Gpr17-regulated cellular processes in oligodendrocyte progenitor cells and underlying myelin dysregulation in the aged mouse cerebrum [86], again supporting a central role for GPR17 in myelination throughout life. 

However, the exact mechanisms at the basis of MTK oligodendrocyte repair and white matter protection remain to be elucidated. 

## 5. Therapeutic Approach Targeting P2 and P2Y-like Receptors in Genetic Models of Postnatal Neurodevelopmental Disorders

**Figure 3:**
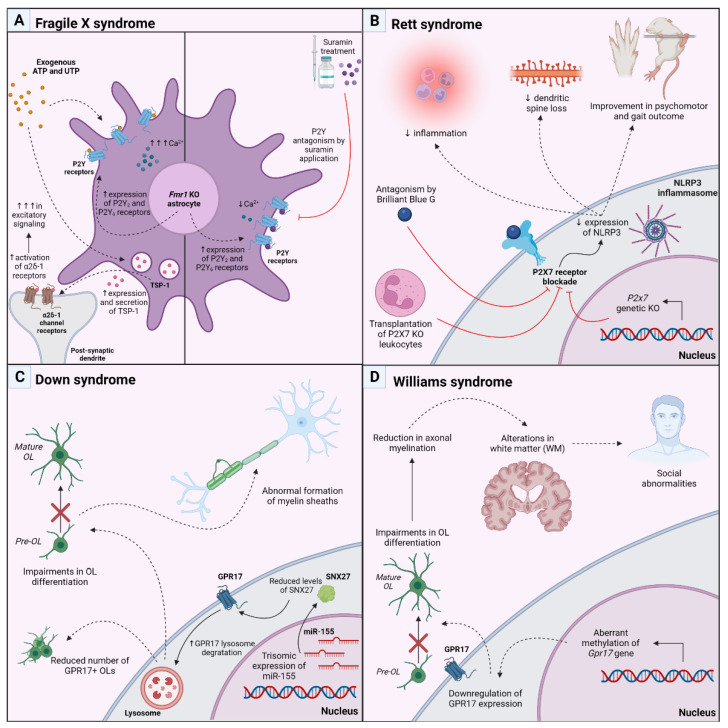
Exemplification scheme of the pathogenic mechanisms leading to the development of some neurodevelopmental disorders, in which a role of P2 or P2-like purinergic receptors has been observed. (**A**). In the Fragile X syndrome, the reduced expression of the Fmr1 gene in cultured astrocytes results in increased expression of P2Y2 and P2Y6 purinergic receptors, which, in turn, leads to augmented intracellular levels of calcium, following the application of exogenous ATP and UTP. In addition, UTP application also induces a higher secretion and expression of TSP-1 glycoprotein, which, by interacting with and activating neuronal α2δ-1 channels, promotes the establishment of a stronger excitatory signaling. On the contrary, P2Y antagonism exerted by suramin prevents intracellular calcium elevations. (**B**). In the *MECP2* KO mice model of Rett syndrome, either the silencing of the expression of *P2x7* gene, the transplantation of *P2X7* KO leukocytes, or the P2X7 receptor blockade obtained from the antagonism by Brilliant Blue G, results in a decrease in inflammation, in a reduction in dendritic spine loss and in an overall improvement in psychomotor outcomes. (**C**). In the Ts65Dn mouse model of Down syndrome, trisomic expression of miR-155 reduces SNX27 protein expression. This was hypothesized to significantly drive GPR17 toward lysosomal degradation which, in turn, is responsible for the observed reduction in GPR17+ cells and for the altered differentiation of oligodendrocytes (OL). (**D**). In Williams syndrome, the aberrant hypomethylation of the *Gpr17* gene downregulates its expression, impairing OL maturation, reducing the physiological axonal myelination and promoting white matter alterations, eventually leading to social and behavioral abnormalities. An upward pointing arrow means an increase, whereas a downward pointing arrow is indicative of a decrease.

The fragile X messenger ribonucleoprotein 1 (*Fmr1*) knockout (KO) mouse is the oldest and one of the most studied genetic mouse models used in autism research. The *Fmr1* gene encodes the fragile X messenger ribonucleoprotein (FMRP), an mRNA-binding protein with a key role in regulating mRNA stability and splicing as well as synaptic plasticity and dendritic transport [87]. In humans, the expansion and hypermethylation of CGG repeat within the *Fmr1* gene, effectively silencing the gene and preventing the expression of the FMRP protein [88], leading to the Fragile X syndrome (FXS), the most common inheritable form of ASD. Similarly, *Fmr1* KO mice display ASD-like behavior, with impairments in social interactions, attention, communication and cognition which are accompanied by synaptic deficits, abnormal dendritic spine morphology and neurotransmission defects [89]. One month of weekly therapy with suramin (20 mg/kg intraperitoneally), started at 9 weeks of age, ameliorated the altered social behavior observed in the *Fmr1* KO mice. In addition, suramin restored the synaptosome structural abnormalities and corrected the altered protein expression of synaptosomal glutamate, endocannabinoid, purinergic and IP3 receptors, complementing C1q, TDP43, ß and amyloid precursor protein (APP) which are present in the mutated mice. When the authors analyzed the metabolomic effects of weekly treatment with suramin or saline in the plasma of Fragile X mice, they identified 20 biochemical pathways associated with symptom improvements in the Fragile X (*Fmr1* knockout) model. Interestingly, there was a generalized increase in gangliosides, phospholipids, and cholesterol metabolites needed for myelin and cell membrane synthesis [90]. Notably, astrocytes are directly involved in the synaptic abnormalities observed in both FXS mouse model and patients: the selective loss of FMRP in astrocytes leads to abnormal dendritic growth and aberrant synapse formation in developing WT neurons [91], whereas WT astrocytes efficiently normalize neuronal alterations in co-culture systems [92]. However, the astrocyte-derived factors impacted by the mutation are largely unknown. In this respect, in cultured astrocytes, the absence of FMRP resulted in elevated expressions of P2Y_2_ and P2Y_6_ purinergic receptors, which, in turn, resulted in higher intracellular calcium responses following the application of exogenous ATP and UTP compared to wildtype cells. In contrast, P2Y antagonism via suramin prevented intracellular calcium elevations. Further, the secretion and expression of the adhesion glycoprotein thrombospondin-1 (TSP-1), which normally promotes the formation of excitatory synapses through its interaction with the neuronal α2δ-1 voltage-gated calcium channel, were both heightened in *Fmr1* KO vs. wildtype astrocytes following UTP application (Figure 3A). This led the authors to speculate that purinergic signaling aberrations contribute to pathologically increased excitatory signaling in Fragile X Syndrome and may necessitate further investigation as a potential therapeutic target for future treatment of FXS [93]. A paper from the same group recently reported evidence of elevated levels of IL-6 and Tenascin C (TNC), a known endogenous ligand of TLR4 that has been shown to induce the expression of pro-inflammatory cytokines such as IL-6 in *Fmr1* KO astrocytes, indicating a potential immune mechanism associated with FXS [94]. However, the stimulation of *Fmr1* KO astrocytes with UDP did not further exacerbate TNC or pSTAT3 levels, a transcription factor linked to the astrocytic production of pro-inflammatory cytokines, suggesting that the purine-mediated activation of these factors was already maximized due to the overexpression of P2Y_2_ and P2Y_6_ receptors.

Rett syndrome (RTT) is another example of monogenic postnatal neurodevelopmental disorder, classified as an autism spectrum disorder, caused by mutations in the X-linked gene encoding methyl-CpG-binding protein 2 (MECP2). The main function of the MECP2 protein is to modulate gene expression by binding methylated CpG dinucleotides by interacting with transcription factors. Clinically, it is characterized by psychomotor regression with a loss of volitional hand use and spoken language, the development of repetitive hand stereotypies and gait impairment. In rodents, RTT has been modeled with a loss of function mutations in MECP2. These mice develop neurological and behavioral phenotypes resembling those observed in RTT patients [95]. In a recently published paper, Garré and co-workers [96] showed that in the *Mecp^2308/Y^* mouse model, there was an upregulation of *P2X7*-positive monocytes and macrophages at the border of the cortical region indicating that the KO of MECP protein predisposed mice to an inflammatory phenotype. Moreover, in the double transgenic *Mecp2308/Y;P2x7r*−/− mice, the ablation of *P2X7* reduced the number of cells positive for the inflammasome maker NLRP3+ and restored the dendritic spine loss observed in the cortex of *Mecp2308/Y* mice. These ameliorations in the inflammatory state and synapse plasticity due to *P2X7R* deficiency were paralleled by an improvement in behavioral outcomes in *Mecp2308/Y* mice. Furthermore, the transplantation of *P2X7*^−^ bone marrow-derived leukocytes in mutated mice, or pharmacological blockade of the receptor by Brillant blue G, recapitulated the beneficial effects of total *P2X7R* depletion on the social behavior and highlighted the positive effects of blocking *P2X7Rs* in *Mecp2308/Y* in peripheral tissues (Figure 3B). Together, these results underscore the contributions of *P2X7Rs* and non-microglial myeloid cells to brain dysfunction in RTT [96].

Recent evidence has highlighted a role for the G protein-coupled P2Y-like receptor GPR17 in neurodevelopmental disorders of genetic origin. After the original report describing GPR17 [74], this receptor has been widely recognized as an important regulator of OPC maturation, both during early development and at adult stages. This receptor responds to both extracellular nucleotides, mainly UDP and UDP–glucose, and cysteinyl–leukotrienes [74], endogenous signaling molecules involved in both inflammatory response and in the repair of CNS lesions. It is highly expressed during the transition from OPCs to immature OLs, but, after this stage, it has to be downregulated to allow cells to complete their maturation toward myelinating OLs [80,97]. Any alteration in this peculiar expression pattern results in myelination defects; marked GPR17 upregulation and/or accumulation of GPR17-expressing cells at the border of demyelinated lesions has been observed in a wide variety of pathological conditions associated with dysmyelination, including patients affected by MS [97,98], traumatic brain injury [99] and congenital leukoencephalopathy [100]. From a neurodevelopmental perspective, GPR17-expressing OPCs are already present in mice at birth, their number increases over time, reaching the highest expression level at P10 before the peak of myelination, and then declines in the adult brain [101].

The first hint of an altered expression of GPR17 in a neurodevelopmental disease came from our observations in the brains of Ts65Dn mice, an animal model of Down Syndrome. In these mice, the trisomic expression of miR-155 leads to reduced levels of sorting nexin 27 (SNX27) [102,103]. SNX27 is a member of a large family of endosomal trafficking proteins which is required for the recycling to the plasma membrane of a number of receptors and ion channels [104]. Of note, we showed that silencing SNX27 significantly increases GPR17 degradation in vitro. In agreement, the number of GPR17-expressing cells was greatly diminished in the postnatal brains of Ts65Dn mice which also showed impaired myelination [105] (Figure 3C).

A very recent publication also reported an alteration of GPR17 expression at the transcriptomic level in the Williams Syndrome (WS), a rare genetic multi-systemic neurodevelopmental disorder caused by a pathological microdeletion of 26–28 genes in the 7q11.23 region. WS is characterized by social abnormalities, such as hyper-sociability, striking social fearlessness, distinct over-friendliness, and excessive empathy, along with poor social judgment ability. These behavior defects are accompanied by a reduction in myelin-related genes, OL numbers, neuronal myelination and by alteration in white matter compared to normally developed control subjects [106,107]. In the study from Trangle and co-workers [108], genome-wide disruption of the methylome has been observed in samples from the frontal cortex of individuals with WS compared to matched controls. Interestingly, gene ontology (GO) analysis revealed that hypomethylated regions/sites of WS patients were enriched in pathways linked to ‘oligodendrocyte specification and differentiation’. In addition, this aberrant hypomethylation in WS individuals led to the transcriptional downregulation of the *GPR17* gene, suggesting a central role for this receptor in the WS-associated phenotype (Figure 3D).

In conclusion, these findings uncover a critical role of GPR17 in the OL defects that are observed in neurodevelopmental disorders and suggest that modulation of its signaling might be a potential therapeutic approach for treating these conditions.

## 6. Conclusions

The evidence discussed here strongly supports a role for P2 receptors and their signaling in NDDs (Table 1).

Specifically, it has been suggested that various kinds of gestational interfering agents resulting in MIA invariably induce increased purine levels in the developing fetal brain. Abnormally elevated purine levels may then act as DAMPs, ultimately leading to an inflammatory response that irreversibly alters physiological CNS development. Depending upon the nature of the interfering agent, exact gestational timing, insult’s duration and/or intensity and the preferential involvement of specific brain areas, the offspring may develop diverse clinical manifestations, ranging from mild-to-serious cognitive impairment, altered social behavior, distinct sensorimotor neurological defects and alterations of specific purinergic pathways or receptors.

Based on both pharmacologic and genetic KO approaches, in animal studies the P2X7 receptor has emerged as one of the purinergic components that most likely contributes to the development of pathological phenotypes in NDDs; more recent evidence also involves the P2Y-like GPR17 receptor.

The pharmacological studies demonstrating an almost complete reversal of NDD symptoms in both animal models and humans are of particular interest. Specifically, the results of the small clinical trial demonstrating astonishing amelioration of symptoms in children with ASDs receiving low-dose suramin [69] open up possibilities for safe and novel disease-modifying therapies.

There are, however, several issues that still needing investigation.

First of all, in all pharmacological studies, symptom reversal proved to be short-lived and highly dependent on continuous drug administration. Drug withdrawal invariably resulted in a return to the pathological phenotype, pointing to the need of chronic lifelong treatments. It would of course be extremely interesting to test whether treatment of mothers during gestation would more permanently reverse/prevent the aberrant neurological phenotype in offspring, thus representing a more definitive resolution for these diseases.

The gestational treatment of mothers at risk would also necessarily require the possibility of early diagnosis, which could be achieved by specifically monitoring ongoing changes in pregnant mothers suspected of being exposed to MIA-inducing agents. Identifying such a patient subpopulation is not an easy task, and, in addition to this, more specific markers would be necessary. In fact, due to lack of specificity, the evaluation of increased purine levels in the blood is not likely to represent a reliable diagnostic/prognostic marker.

Nevertheless, we believe that studies in this field deserve strong attention and support; future attempts should focus on more receptor-specific ligands [109] and/or on drugs such as MTK [83,84,85], that are already approved for human use and have been already validated in animal models as safe and effective neuroprotective agents.

## Figures and Tables

**Table 1:** Summary table of the P2 and P2-like receptors’ involvement in the etiopathogenesis of several neurodevelopmental disorders.

Receptor	Implicated Diseases	Therapeutic Targets or Receptor Engagement
P2Y2	Autism spectrum disorders (ASDs)	Upregulated in the blood of children with ASDs [40] Reduced expression in the Poly(I:C)-induced ASD mouse model [61]
	Fragile X syndrome (FXS)	Upregulated in cultured *Fmr1* KO astrocytes [93]
P2Y6	Autism spectrum disorders (ASDs)	Upregulated in the blood of children with ASDs [40]
	Fragile X syndrome (FXS)	Upregulated in cultured *Fmr1* KO astrocytes [93]
P2X4	Schizophrenia (SCZ)	mRNA significantly increased in the dorsolateral prefrontal cortex of schizophrenic subjects [43]
P2X5	Schizophrenia (SCZ)	mRNA significantly increased in the dorsolateral prefrontal cortex of schizophrenic subjects [43]
P2X7	Autism spectrum disorders (ASDs)	Downregulated in the blood of children with ASDs [40] *P2X7* KO abolished the ASDs related abnormalities induced by Poly(I:C) treatment during pregnancy [66] Maternal and the early postnatal treatment with the selective antagonist JNJ47965567 in MIA mouse models of ASDs induced improvements compare to untreated mice [66] JNJ47965567 administration in early adulthood (P65) in MIA offspring prevented the onset of autism-like behaviors [67]
	Schizophrenia (SCZ)	Increased in the dorsolateral prefrontal cortex of schizophrenic subjects [43] Involved in the appearance of schizophrenia-like behaviors and in impairments in dendritic outgrowth in hippocampal neurons in the MIA model [68]
	Rett syndrome (RTT)	Upregulated in monocytes and macrophages at the border of the cortical region of *Mecp2308/Y* mouse model [96] In the double transgenic *Mecp2308/Y;P2x7r*−/− mice has been noted a reduction in the NLRP3+ cells, a restoration of the cortical dendritic spine loss and behavioral improvements compared to MECP2 KO mice [96] Both the transplantation of *P2X7* KO bone marrow-derived leukocytes in WT mice and the pharmacological blockade of P2X7 receptor by Brilliant Blue G have been observed to induce beneficial effects in *MECP2* KO mice [96]
GPR17	Periventricular Leukomalacia (PVL)	Significantly upregulated in early postnatal PVL rats compared to controls [77] In vivo silencing in PVL rat models induces a significant increase in the formation of myelin sheaths as well as thicker myelin [77]
	Down syndrome	Significantly reduction in the number of GPR17+ cells in the brain of Ts65Dn mice [105]
	Williams syndrome (WS)	Hypomethylation and transcriptional downregulation of *Gpr17* gene [108]

## Data Availability

Not applicable.

## References

[1] Thapar A., Cooper M., Rutter M. (2017). Neurodevelopmental Disorders. Lancet Psychiatry.

[2] Parenti I., Rabaneda L.G., Schoen H., Novarino G. (2020). Neurodevelopmental Disorders: From Genetics to Functional Pathways. Trends. Neurosci..

[3] Shook L.L., Sullivan E.L., Lo J.O., Perlis R.H., Edlow A.G. (2022). COVID-19 in Pregnancy: Implications for Fetal Brain Development. Trends. Mol. Med..

[4] Jiang X., Nardelli J. (2016). Cellular and Molecular Introduction to Brain Development. Neurobiol. Dis..

[5] Silbereis J.C., Pochareddy S., Zhu Y., Li M., Sestan N. (2016). The Cellular and Molecular Landscapes of the Developing Human Central Nervous System. Neuron.

[6] Cunha R.A. (2001). Adenosine as a Neuromodulator and as a Homeostatic Regulator in the Nervous System: Different Roles, Different Sources and Different Receptors. Neurochem. Int..

[7] Abbracchio M.P., Ceruti S. (2006). Roles of P2 Receptors in Glial Cells: Focus on Astrocytes. Purinergic. Signal..

[8] Fumagalli M., Lecca D., Abbracchio M.P., Ceruti S. (2017). Pathophysiological Role of Purines and Pyrimidines in Neurodevelopment: Unveiling New Pharmacological Approaches to Congenital Brain Diseases. Front. Pharmacol..

[9] Boison D., Singer P., Shen H.Y., Feldon J., Yee B.K. (2012). Adenosine Hypothesis of Schizophrenia—Opportunities for Pharmacotherapy. Neuropharmacology.

[10] Heine C., Sygnecka K., Franke H. (2016). Purines in Neurite Growth and Astroglia Activation. Neuropharmacology.

[11] Alçada-Morais S., Gonçalves N., Moreno-Juan V., Andres B., Ferreira S., Marques J.M., Magalhães J., Rocha J.M.M., Xu X., Partidário M. (2021). Adenosine A2A Receptors Contribute to the Radial Migration of Cortical Projection Neurons through the Regulation of Neuronal Polarization and Axon Formation. Cereb. Cortex..

[12] Silva C.G., Métin C., Fazeli W., Machado N.J., Darmopil S., Launay P.S., Ghestem A., Nesa M.P., Bassot E., Szabó E. (2013). Adenosine Receptor Antagonists Including Caffeine Alter Fetal Brain Development in Mice. Sci. Transl. Med..

[13] Gomez-Castro F., Zappettini S., Pressey J.C., Silva C.G., Russeau M., Gervasi N., Figueiredo M., Montmasson C., Renner M., Canas P.M. (2021). Convergence of Adenosine and GABA Signaling for Synapse Stabilization during Development. Science.

[14] Xu X., Beleza R.O., Gonçalves F.Q., Valbuena S., Alçada-Morais S., Gonçalves N., Magalhães J., Rocha J.M.M., Ferreira S., Figueira A.S.G. (2022). Adenosine A2A Receptors Control Synaptic Remodeling in the Adult Brain. Sci. Rep..

[15] Coppi E., Dettori I., Cherchi F., Bulli I., Venturini M., Pedata F., Pugliese A.M. (2021). New Insight into the Role of Adenosine in Demyelination, Stroke and Neuropathic Pain. Front. Pharmacol..

[16] Oliveira Á., Illes P., Ulrich H. (2016). Purinergic Receptors in Embryonic and Adult Neurogenesis. Neuropharmacology.

[17] Ulrich H., Abbracchio M.P., Burnstock G. (2012). Extrinsic Purinergic Regulation of Neural Stem/Progenitor Cells: Implications for CNS Development and Repair. Stem. Cell. Rev..

[18] Cserép C., Schwarcz A.D., Pósfai B., László Z.I., Kellermayer A., Környei Z., Kisfali M., Nyerges M., Lele Z., Katona I. (2022). Microglial Control of Neuronal Development via Somatic Purinergic Junctions. Cell. Rep..

[19] Buffo A., Rolando C., Ceruti S. (2010). Astrocytes in the Damaged Brain: Molecular and Cellular Insights into Their Reactive Response and Healing Potential. Biochem. Pharmacol..

[20] Fumagalli M., Lecca D., Abbracchio M.P. (2016). CNS Remyelination as a Novel Reparative Approach to Neurodegenerative Diseases: The Roles of Purinergic Signaling and the P2Y-like Receptor GPR17. Neuropharmacology.

[21] Fields R.D., Stevens B. (2000). ATP: An Extracellular Signaling Molecule between Neurons and Glia. Trends. Neurosci..

[22] Suyama S., Sunabori T., Kanki H., Sawamoto K., Gachet C., Koizumi S., Okano H. (2012). Purinergic Signaling Promotes Proliferation of Adult Mouse Subventricular Zone Cells. J. Neurosci..

[23] Boccazzi M., Rolando C., Abbracchio M.P., Buffo A., Ceruti S. (2014). Purines Regulate Adult Brain Subventricular Zone Cell Functions: Contribution of Reactive Astrocytes. Glia.

[24] Boda E., Nato G., Buffo A. (2017). Emerging Pharmacological Approaches to Promote Neurogenesis from Endogenous Glial Cells. Biochem. Pharmacol..

[25] del Puerto A., Díaz-Hernández J.I., Tapia M., Gomez-Villafuertes R., Benitez M.J., Zhang J., Miras-Portugal M.T., Wandosell F., Díaz-Hernández M., Garrido J.J. (2012). Adenylate Cyclase 5 Coordinates the Action of ADP, P2Y1, P2Y13 and ATP-Gated P2X7 Receptors on Axonal Elongation. J. Cell. Sci..

[26] Sebastián-Serrano A., Engel T., De Diego-García L., Olivos-Oré L.A., Arribas-Blázquez M., Martínez-Frailes C., Pérez-Díaz C., Luis Millán J., Artalejo A.R., Miras-Portugal M.T. (2016). Neurodevelopmental Alterations and Seizures Developed by Mouse Model of Infantile Hypophosphatasia Are Associated with Purinergic Signalling Deregulation. Hum. Mol. Genet..

[27] Rodrigues R.J., Marques J.M., Cunha R.A. (2019). Purinergic Signalling and Brain Development. Semin. Cell. Dev. Biol..

[28] Guo M., Xie P., Liu S., Luan G., Li T. (2023). Epilepsy and Autism Spectrum Disorder (ASD): The Underlying Mechanisms and Therapy Targets Related to Adenosine. Curr. Neuropharmacol..

[29] Singer P., Yee B.K. (2023). The Adenosine Hypothesis of Schizophrenia into Its Third Decade: From Neurochemical Imbalance to Early Life Etiological Risks. Front. Cell. Neurosci..

[30] Pasquini S., Contri C., Merighi S., Gessi S., Borea P.A., Varani K., Vincenzi F. (2022). Adenosine Receptors in Neuropsychiatric Disorders: Fine Regulators of Neurotransmission and Potential Therapeutic Targets. Int. J. Mol. Sci..

[31] Pareek V., Pedley A.M., Benkovic S.J. (2021). Human de Novo Purine Biosynthesis. Crit. Rev. Biochem. Mol. Biol..

[32] Lesch M., Nyhan W.L. (1964). A Familial Disorder of Uric Acid Metabolism and Central Nervous System Function. Am. J. Med..

[33] Harris J.C. (2018). Lesch–Nyhan Syndrome and Its Variants. Curr. Opin. Psychiatry.

[34] Nyhan W.L., James J.A., Teberg A.J., Sweetman L., Nelson L.G. (1969). A New Disorder of Purine Metabolism with Behavioral Manifestations. J. Pediatr..

[35] Gevi F., Zolla L., Gabriele S., Persico A.M. (2016). Urinary Metabolomics of Young Italian Autistic Children Supports Abnormal Tryptophan and Purine Metabolism. Mol. Autism..

[36] Bitar T., Mavel S., Emond P., Nadal-Desbarats L., Lefèvre A., Mattar H., Soufia M., Blasco H., Vourc’h P., Hleihel W. (2018). Identification of Metabolic Pathway Disturbances Using Multimodal Metabolomics in Autistic Disorders in a Middle Eastern Population. J. Pharm. Biomed. Anal..

[37] Kurochkin I., Khrameeva E., Tkachev A., Stepanova V., Vanyushkina A., Stekolshchikova E., Li Q., Zubkov D., Shichkova P., Halene T. (2019). Metabolome Signature of Autism in the Human Prefrontal Cortex. Commun. Biol..

[38] Liang Y., Ke X., Xiao Z., Zhang Y., Chen Y., Li Y., Wang Z., Lin L., Yao P., Lu J. (2020). Untargeted Metabolomic Profiling Using UHPLC-QTOF/MS Reveals Metabolic Alterations Associated with Autism. Biomed. Res. Int..

[39] Mussap M., Siracusano M., Noto A., Fattuoni C., Riccioni A., Rajula H.S.R., Fanos V., Curatolo P., Barberini L., Mazzone L. (2020). The Urine Metabolome of Young Autistic Children Correlates with Their Clinical Profile Severity. Metabolites.

[40] Dai S., Lin J., Hou Y., Luo X., Shen Y., Ou J. (2023). Purine Signaling Pathway Dysfunction in Autism Spectrum Disorders: Evidence from Multiple Omics Data. Front. Mol. Neurosci..

[41] Torres R.J., Prior C., Garcia M.G., Puig J.G. (2016). A Review of the Implication of Hypoxanthine Excess in the Physiopathology of Lesch–Nyhan Disease. Nucleosides Nucleotides Nucleic Acids.

[42] Kristóf Z., Baranyi M., Tod P., Mut-Arbona P., Demeter K., Bitter I., Sperlágh B. (2022). Elevated Serum Purine Levels in Schizophrenia: A Reverse Translational Study to Identify Novel Inflammatory Biomarkers. Int. J. Neuropsychopharmacol..

[43] Alnafisah R., Lundh A., Asah S.M., Hoeflinger J., Wolfinger A., Hamoud A.-r., McCullumsmith R.E., O’Donovan S.M. (2022). Altered Purinergic Receptor Expression in the Frontal Cortex in Schizophrenia. Schizophrenia.

[44] Di Virgilio F., Ceruti S., Bramanti P., Abbracchio M.P. (2009). Purinergic Signalling in Inflammation of the Central Nervous System. Trends. Neurosci..

[45] Rodrigues R.J., Tomé A.R., Cunha R.A. (2015). ATP as a Multi-Target Danger Signal in the Brain. Front. Neurosci..

[46] Naviaux J.C., Schuchbauer M.A., Li K., Wang L., Risbrough V.B., Powell S.B., Naviaux R.K. (2014). Reversal of Autism-like Behaviors and Metabolism in Adult Mice with Single-Dose Antipurinergic Therapy. Transl. Psychiatry.

[47] Minakova E., Warner B.B. (2018). Maternal Immune Activation, Central Nervous System Development and Behavioral Phenotypes. Birth. Defects. Res..

[48] Selten J.-P., Morgan V.A. (2010). Prenatal Exposure to Influenza and Major Affective Disorder. Bipolar. Disord..

[49] Selten J.P., Termorshuizen F. (2017). The Serological Evidence for Maternal Influenza as Risk Factor for Psychosis in Offspring Is Insufficient: Critical Review and Meta-Analysis. Schizophr. Res..

[50] Knuesel I., Chicha L., Britschgi M., Schobel S.A., Bodmer M., Hellings J.A., Toovey S., Prinssen E.P., Pharma Research R., Development E. (2014). Maternal Immune Activation and Abnormal Brain Development across CNS Disorders. Nat. Publ. Group.

[51] Meyer U. (2014). Prenatal Poly(I:C) Exposure and Other Developmental Immune Activation Models in Rodent Systems. Biol. Psychiatry.

[52] Giovanoli S., Engler H., Engler A., Richetto J., Voget M., Willi R., Winter C., Riva M.A., Mortensen P.B., Schedlowski M. (2013). Stress in Puberty Unmasks Latent Neuropathological Consequences of Prenatal Immune Activation in Mice. Science.

[53] Bucknor M.C., Gururajan A., Dale R.C., Hofer M.J. (2022). A Comprehensive Approach to Modeling Maternal Immune Activation in Rodents. Front. Neurosci..

[54] Woods R.M., Lorusso J.M., Potter H.G., Neill J.C., Glazier J.D., Hager R. (2021). Maternal Immune Activation in Rodent Models: A Systematic Review of Neurodevelopmental Changes in Gene Expression and Epigenetic Modulation in the Offspring Brain. Neurosci. Biobehav. Rev..

[55] Garay P.A., Hsiao E.Y., Patterson P.H., McAllister A.K. (2013). Maternal Immune Activation Causes Age- and Region-Specific Changes in Brain Cytokines in Offspring throughout Development. Brain Behav. Immun..

[56] Colucci F., Boulenouar S., Kieckbusch J., Moffett A. (2011). How Does Variability of Immune System Genes Affect Placentation?. Placenta.

[57] Bergdolt L., Dunaevsky A. (2019). Brain Changes in a Maternal Immune Activation Model of Neurodevelopmental Brain Disorders. Prog. Neurobiol..

[58] Solek C.M., Farooqi N., Verly M., Lim T.K., Ruthazer E.S., Doi D. (2018). Maternal Immune Activation in Neurodevelopmental Disorders. Dev. Dyn..

[59] Haddad F.L., Patel S.V., Schmid S. (2020). Maternal Immune Activation by Poly I:C as a Preclinical Model for Neurodevelopmental Disorders: A Focus on Autism and Schizophrenia. Neurosci. Biobehav. Rev..

[60] Reisinger S., Khan D., Kong E., Berger A., Pollak A., Pollak D.D. (2015). The Poly(I:C)-Induced Maternal Immune Activation Model in Preclinical Neuropsychiatric Drug Discovery. Pharmacol. Ther..

[61] Naviaux R.K., Zolkipli Z., Wang L., Nakayama T., Naviaux J.C., Le T.P., Schuchbauer M.A., Rogac M., Tang Q., Dugan L.L. (2013). Antipurinergic Therapy Corrects the Autism-like Features in the Poly(IC) Mouse Model. PLoS ONE.

[62] Bhattacharya A., Jones D.N.C. (2018). Emerging Role of the P2X7-NLRP3-IL1β Pathway in Mood Disorders. Psychoneuroendocrinology.

[63] Sperlágh B., Vizi E.S., Wirkner K., Illes P. (2006). P2X7 Receptors in the Nervous System. Prog. Neurobiol..

[64] Di Virgilio F., Sarti A.C., Coutinho-Silva R. (2020). Purinergic Signaling, DAMPs, and Inflammation. Am. J. Physiol.-Cell Physiol..

[65] Oliveira-Giacomelli Á., Petiz L.L., Andrejew R., Turrini N., Silva J.B., Sack U., Ulrich H. (2021). Role of P2X7 Receptors in Immune Responses during Neurodegeneration. Front. Cell. Neurosci..

[66] Horváth G., Otrokocsi L., Beko K., Baranyi M., Kittel Á., Fritz-Ruenes P.A., Sperlágh B. (2019). P2X7 Receptors Drive Poly(I:C) Induced Autism-like Behavior in Mice. J. Neurosci..

[67] Szabó D., Tod P., Gölöncsér F., Román V., Lendvai B., Otrokocsi L., Sperlágh B. (2022). Maternal P2X7 Receptor Inhibition Prevents Autism-like Phenotype in Male Mouse Offspring through the NLRP3-IL-1β Pathway. Brain Behav. Immun..

[68] Mut-Arbona P., Huang L., Baranyi M., Tod P., Iring A., Calzaferri F., de Los Ríos C., Sperlágh B. (2023). Dual Role of the P2X7 Receptor in Dendritic Outgrowth during Physiological and Pathological Brain Development. J. Neurosci..

[69] Naviaux R.K., Curtis B., Li K., Naviaux J.C., Bright A.T., Reiner G.E., Westerfield M., Goh S., Alaynick W.A., Wang L. (2017). Low-Dose Suramin in Autism Spectrum Disorder: A Small, Phase I/II, Randomized Clinical Trial. Ann. Clin. Transl. Neurol..

[70] Passera S., Boccazzi M., Bokobza C., Faivre V., Mosca F., Van Steenwinckel J., Fumagalli M., Gressens P., Fleiss B. (2021). Therapeutic Potential of Stem Cells for Preterm Infant Brain Damage: Can We Move from the Heterogeneity of Preclinical and Clinical Studies to Established Therapeutics?. Biochem. Pharmacol..

[71] Chawanpaiboon S., Vogel J.P., Moller A.-B., Lumbiganon P., Petzold M., Hogan D., Landoulsi S., Jampathong N., Kongwattanakul K., Laopaiboon M. (2019). Global, Regional, and National Estimates of Levels of Preterm Birth in 2014: A Systematic Review and Modelling Analysis. Lancet Glob. Health.

[72] Vanes L.D., Murray R.M., Nosarti C. (2022). Adult Outcome of Preterm Birth: Implications for Neurodevelopmental Theories of Psychosis. Schizophr. Res..

[73] Favrais G., Bokobza C., Saliba E., Chalon S., Gressens P. (2022). Alteration of the Oligodendrocyte Lineage Varies According to the Systemic Inflammatory Stimulus in Animal Models That Mimic the Encephalopathy of Prematurity. Front. Physiol..

[74] Ciana P., Fumagalli M., Trincavelli M.L., Verderio C., Rosa P., Lecca D., Ferrario S., Parravicini C., Capra V., Gelosa P. (2006). The Orphan Receptor GPR17 Identified as a New Dual Uracil Nucleotides/Cysteinyl-Leukotrienes Receptor. EMBO J..

[75] Mao F.-X., Li W.-J., Chen H.-J., Qian L.-H., Buzby J.S. (2012). Periventricular Leukomalacia Long-Term Prognosis May Be Improved by Treatment with UDP-Glucose, GDNF, and Memantine in Neonatal Rats. Brain Res..

[76] Li W.J., Mao F.X., Chen H.J., Qian L.H., Buzby J.S. (2015). Treatment with UDP-Glucose, GDNF, and Memantine Promotes SVZ and White Matter Self-Repair by Endogenous Glial Progenitor Cells in Neonatal Rats with Ischemic PVL. Neuroscience.

[77] He L., Yang H., Feng J., Wei T., Huang Y., Zhang X., Wang Z. (2021). Knockdown of G Protein-Coupled Receptor-17 (GPR17) Facilitates the Regeneration and Repair of Myelin Sheath Post-Periventricular Leukomalacia (PVL). Bioengineered.

[78] Favrais G., van de Looij Y., Fleiss B., Ramanantsoa N., Bonnin P., Stoltenburg-Didinger G., Lacaud A., Saliba E., Dammann O., Gallego J. (2011). Systemic Inflammation Disrupts the Developmental Program of White Matter. Ann. Neurol..

[79] Boccazzi M., Van Steenwinckel J., Schang A.L., Faivre V., Le Charpentier T., Bokobza C., Csaba Z., Verderio C., Fumagalli M., Mani S. (2021). The Immune-Inflammatory Response of Oligodendrocytes in a Murine Model of Preterm White Matter Injury: The Role of TLR3 Activation. Cell Death Dis..

[80] Fumagalli M., Daniele S., Lecca D., Lee P.R., Parravicini C., Fields R.D., Rosa P., Antonucci F., Verderio C., Trincavelli M.L. (2011). Phenotypic Changes, Signaling Pathway, and Functional Correlates of GPR17-Expressing Neural Precursor Cells during Oligodendrocyte Differentiation. J. Biol. Chem..

[81] Fratangeli A., Parmigiani E., Fumagalli M., Lecca D., Benfante R., Passafaro M., Buffo A., Abbracchio M.P., Rosa P. (2013). The Regulated Expression, Intracellular Trafficking, and Membrane Recycling of the P2Y-like Receptor GPR17 in Oli-Neu Oligodendroglial Cells. J. Biol. Chem..

[82] Köse M., Ritter K., Thiemke K., Gillard M., Kostenis E., Müller C.E. (2014). Development of [^3^H]2-Carboxy-4,6-Dichloro-1 H-Indole-3-Propionic Acid ([^3^H]PSB-12150): A Useful Tool for Studying GPR17. ACS Med. Chem. Lett..

[83] Gelosa P., Bonfanti E., Castiglioni L., Delgado-Garcia J.M., Gruart A., Fontana L., Gotti M., Tremoli E., Lecca D., Fumagalli M. (2019). Improvement of Fiber Connectivity and Functional Recovery after Stroke by Montelukast, an Available and Safe Anti-Asthmatic Drug. Pharmacol. Res..

[84] Bonfanti E., Bonifacino T., Raffaele S., Milanese M., Morgante E., Bonanno G., Abbracchio M.P., Fumagalli M. (2020). Abnormal Upregulation of GPR17 Receptor Contributes to Oligodendrocyte Dysfunction in SOD1 G93A Mice. Int. J. Mol. Sci..

[85] Marschallinger J., Schäffner I., Klein B., Gelfert R., Rivera F.J., Illes S., Grassner L., Janssen M., Rotheneichner P., Schmuckermair C. (2015). Structural and Functional Rejuvenation of the Aged Brain by an Approved Anti-Asthmatic Drug. Nat. Commun..

[86] Rivera A.D., Pieropan F., Chacon-De-La-Rocha I., Lecca D., Abbracchio M.P., Azim K., Butt A.M. (2021). Functional Genomic Analyses Highlight a Shift in Gpr17-Regulated Cellular Processes in Oligodendrocyte Progenitor Cells and Underlying Myelin Dysregulation in the Aged Mouse Cerebrum. Aging Cell.

[87] Prashad S., Gopal P.P. (2021). RNA-Binding Proteins in Neurological Development and Disease. RNA Biol..

[88] Pieretti M., Zhang F., Fu Y.H., Warren S.T., Oostra B.A., Caskey C.T., Nelson D.L. (1991). Absence of Expression of the FMR-1 Gene in Fragile X Syndrome. Cell.

[89] Kazdoba T.M., Leach P.T., Silverman J.L., Crawley J.N. (2014). Modeling Fragile X Syndrome in the Fmr1 Knockout Mouse. Intractable Rare. Dis. Res..

[90] Naviaux J.C., Wang L., Li K., Bright A.T., Alaynick W.A., Williams K.R., Powell S.B., Naviaux R.K. (2015). Antipurinergic Therapy Corrects the Autism-like Features in the Fragile X (Fmr1 Knockout) Mouse Model. Mol. Autism..

[91] Hodges J.L., Yu X., Gilmore A., Bennett H., Tjia M., Perna J.F., Chen C.C., Li X., Lu J., Zuo Y. (2017). Astrocytic Contributions to Synaptic and Learning Abnormalities in a Mouse Model of Fragile X Syndrome. Biol. Psychiatry.

[92] Jacobs S., Doering L.C. (2010). Astrocytes Prevent Abnormal Neuronal Development in the Fragile X Mouse. J. Neurosci..

[93] Reynolds K.E., Wong C.R., Scott A.L. (2021). Astrocyte-Mediated Purinergic Signaling Is Upregulated in a Mouse Model of Fragile X Syndrome. Glia.

[94] Krasovska V., Doering L.C. (2018). Regulation of IL-6 Secretion by Astrocytes via TLR4 in the Fragile X Mouse Model. Front. Mol. Neurosci..

[95] Bach S., Shovlin S., Moriarty M., Bardoni B., Tropea D. (2021). Rett Syndrome and Fragile X Syndrome: Different Etiology with Common Molecular Dysfunctions. Front. Cell. Neurosci..

[96] Garré J.M., Silva H.M., Lafaille J.J., Yang G. (2020). P2X7 Receptor Inhibition Ameliorates Dendritic Spine Pathology and Social Behavioral Deficits in Rett Syndrome Mice. Nat. Commun..

[97] Chen Y., Wu H., Wang S., Koito H., Li J., Ye F., Hoang J., Escobar S.S., Gow A., Arnett H.A. (2009). The Oligodendrocyte-Specific G Protein-Coupled Receptor GPR17 Is a Cell-Intrinsic Timer of Myelination. Nat. Neurosci..

[98] Angelini J., Marangon D., Raffaele S., Lecca D., Abbracchio M.P. (2021). The Distribution of Gpr17-Expressing Cells Correlates with White Matter Inflammation Status in Brain Tissues of Multiple Sclerosis Patients. Int. J. Mol. Sci..

[99] Franke H., Parravicini C., Lecca D., Zanier E.R., Heine C., Bremicker K., Fumagalli M., Rosa P., Longhi L., Stocchetti N. (2013). Changes of the GPR17 Receptor, a New Target for Neurorepair, in Neurons and Glial Cells in Patients with Traumatic Brain Injury. Purinergic. Signal..

[100] Satoh J.I., Kino Y., Yanaizu M., Tosaki Y., Sakai K., Ishida T., Saito Y. (2017). Expression of GPR17, a Regulator of Oligodendrocyte Differentiation and Maturation, in Nasu-Hakola Disease Brains. Intractable Rare. Dis. Res..

[101] Boda E., Viganò F., Rosa P., Fumagalli M., Labat-Gest V., Tempia F., Abbracchio M.P., Dimou L., Buffo A. (2011). The GPR17 Receptor in NG2 Expressing Cells: Focus on in Vivo Cell Maturation and Participation in Acute Trauma and Chronic Damage. Glia.

[102] Wang X., Huang T., Zhao Y., Zheng Q., Thompson R.C., Bu G., Zhang Y., Hong W., Xu H. (2014). Sorting Nexin 27 Regulates Aβ Production through Modulating γ-Secretase Activity. Cell Rep..

[103] Wang X., Zhao Y., Zhang X., Badie H., Zhou Y., Mu Y., Loo L.S., Cai L., Thompson R.C., Yang B. (2013). Loss of Sorting Nexin 27 Contributes to Excitatory Synaptic Dysfunction by Modulating Glutamate Receptor Recycling in Down’s Syndrome. Nat. Med..

[104] Chandra M., Kendall A.K., Jackson L.P. (2021). Toward Understanding the Molecular Role of SNX27/Retromer in Human Health and Disease. Front. Cell Dev. Biol..

[105] Meraviglia V., Ulivi A.F., Boccazzi M., Valenza F., Fratangeli A., Passafaro M., Lecca D., Stagni F., Giacomini A., Bartesaghi R. (2016). SNX27, a Protein Involved in down Syndrome, Regulates GPR17 Trafficking and Oligodendrocyte Differentiation. Glia.

[106] Nir A., Barak B. (2021). White Matter Alterations in Williams Syndrome Related to Behavioral and Motor Impairments. Glia.

[107] Barak B., Zhang Z., Liu Y., Nir A., Trangle S.S., Ennis M., Levandowski K.M., Wang D., Quast K., Boulting G.L. (2019). Neuronal Deletion of Gtf2i, Associated with Williams Syndrome, Causes Behavioral and Myelin Alterations Rescuable by a Remyelinating Drug. Nat. Neurosci..

[108] Trangle S.S., Rosenberg T., Parnas H., Levy G., Bar E., Marco A., Barak B. (2023). In Individuals with Williams Syndrome, Dysregulation of Methylation in Non-Coding Regions of Neuronal and Oligodendrocyte DNA Is Associated with Pathology and Cortical Development. Mol. Psychiatry.

[109] Calleri E., Ceruti S., Cristalli G., Martini C., Temporini C., Parravicini C., Volpini R., Daniele S., Caccialanza G., Lecca D. (2010). Frontal Affinity Chromatography-Mass Spectrometry Useful for Characterization of New Ligands for GPR17 Receptor. J. Med. Chem..

